# Ecological consequences of long-term browning in lakes

**DOI:** 10.1038/srep18666

**Published:** 2015-12-22

**Authors:** Craig E. Williamson, Erin P. Overholt, Rachel M. Pilla, Taylor H. Leach, Jennifer A. Brentrup, Lesley B. Knoll, Elizabeth M. Mette, Robert E. Moeller

**Affiliations:** 1 Department of Biology, Miami University, Oxford, OH 45056, USA; 2 Lacawac Sanctuary, Lake Ariel, PA 18436, USA

## Abstract

Increases in terrestrially-derived dissolved organic matter (DOM) have led to the browning of inland waters across regions of northeastern North America and Europe. Short-term experimental and comparative studies highlight the important ecological consequences of browning. These range from transparency-induced increases in thermal stratification and oxygen (O_2_) depletion to changes in pelagic food web structure and alteration of the important role of inland waters in the global carbon cycle. However, multi-decadal studies that document the net ecological consequences of long-term browning are lacking. Here we show that browning over a 27 year period in two lakes of differing transparency resulted in fundamental changes in vertical habitat gradients and food web structure, and that these responses were stronger in the more transparent lake. Surface water temperatures increased by 2–3 °C in both lakes in the absence of any changes in air temperature. Water transparency to ultraviolet (UV) radiation showed a fivefold decrease in the more transparent lake. The primary zooplankton grazers decreased, and in the more transparent lake were largely replaced by a two trophic level zooplankton community. These findings provide new insights into the net effects of the complex and contrasting mechanisms that underlie the ecosystem consequences of browning.

The causes of the browning of inland waters have been shown to be related to a combination of recovery from anthropogenic acidification^1^, increases in precipitation^2^,^3^, and other factors such as changes in land use and DOM-iron interactions^4^,^5^. In some regions where browning is prevalent such as northeastern North America, up to a 71% increase in very heavy precipitation events has been reported in the past 5 decades^6^. Browning results from increases in not only dissolved organic carbon (DOC) concentration^1^, but also DOC color (DOC-specific absorbance at 320 nm, a*_320nm_)^7^, and is often accompanied by increasing water pH^1^.

Prior short-term experimental, comparative, and modeling studies suggest that long-term browning will alter vertical habitat gradients in pelagic ecosystems with important ecological consequences^8^. Two contrasting mechanisms have been hypothesized to underlie these ecological consequences of browning: (1) the provision of ecosystem subsidies in the form of nutrients or fixed carbon, and (2) the effects of shading^8^,^9^. Terrestrial DOM can provide nutrient subsidies that enhance zooplankton production^10^, while the shading effects of DOM inhibit photosynthesis and consequently reduce food supply for higher trophic levels including zooplankton and fish^11^. Simultaneous increases in pH and decreases in UV exposure may also alter consumer-resource interactions during browning^12^. All of these mechanisms can lead to reductions in water transparency to UV and photosynthetically active radiation (PAR, 400–700 nm) that have the potential to influence vertical habitat gradients, food web structure, and ultimately ecosystem services. Accumulating evidence suggests that the effects of browning are context dependent and vary with the initial color of the lake. Clear-water oligotrophic lakes are hypothesized to be more sensitive to browning than are lakes with initially higher DOM^13^. Modeling and comparative studies even suggest a unimodal response wherein clear-water oligotrophic lakes respond to browning in the opposite way as do already brown lakes^14^. But do the results of these short-term experimental, comparative, and modeling studies accurately forecast the response of lakes to long-term browning? How do the ecological consequences differ in clear-water versus brown-water lakes? And are important water transparency related phenological events such the UV or PAR clear-water phases^15^ altered during browning? The multi-decadal observational studies that are necessary to resolve these questions and their implications for important ecosystem services are lacking. Here we present 27 years of data on two small lakes of differing transparency that demonstrate changes in the vertical habitat gradients and food web structure during browning that are especially strong in the clearer lake.

The two small study lakes are located in protected watersheds in northeastern Pennsylvania, USA, a region that has experienced both the decrease in acid deposition as well as the increase in precipitation characteristic of regions where lakes are browning^2^,^3^,^6^,^16^. Lake Lacawac is a 21 ha dystrophic brown-water lake (DOC = 5–6 mg C L^−1^) with a maximum depth of 13 m and a catchment located within a sanctuary preserve, while Lake Giles is a 48 ha oligotrophic clear-water lake (DOC = 1–2 mg C L^−1^) with a maximum depth of 23 m and a catchment located within a privately owned and well-protected watershed. These lakes are near and below the global median DOC concentration of 5.7 mg C L^−1^ for lakes^17^. Both are seepage lakes with no tributary streams or rivers other than a small spring on the shore of each lake. There have been no changes in land use or land cover in the privately owned and well-protected catchments, thus leaving only atmospheric or meteorological factors as potential agents of external forcing in these lakes.

## Results

**Figure 1:**
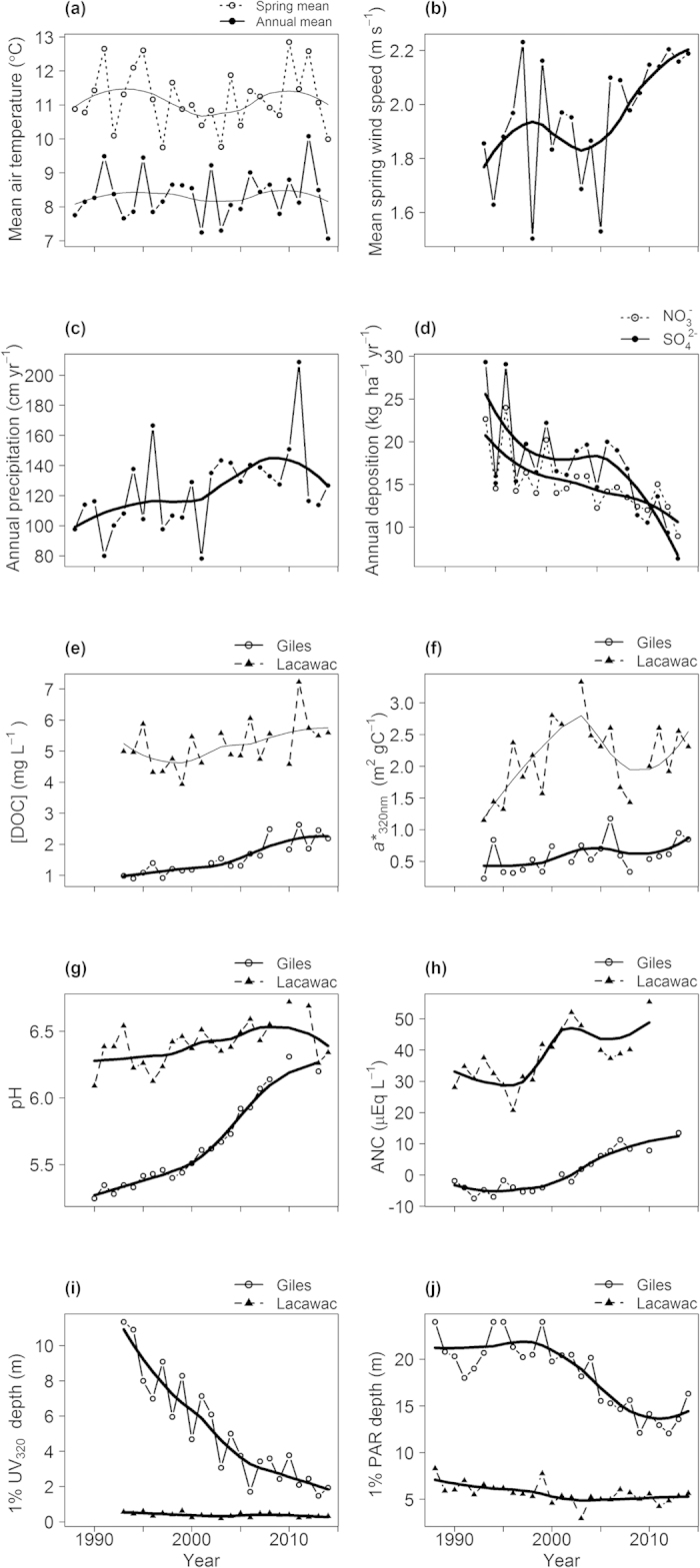
Interannual changes in meteorological and limnological variables during the study period. (**a**) Mean spring (March – mid-July date of sampling) and annual air temperature (°C). (**b**) Average spring (March – April) wind speed (m s^−1^). (**c**) Annual precipitation (cm yr^−1^). (**d**) Annual atmospheric deposition of NO_3_^−^ and SO_4_^2−^ (kg ha^−1^ yr^−1^). (**e**) DOC concentration (mg L^−1^) in the surface waters. (**f** ) DOC-specific absorbance at 320 nm (a*320nm, m^2^ (g C)^−1^) in the surface waters, a measure of DOC color. (**g**) Surface water pH. (**h**) Surface water acid-neutralizing capacity (ANC, μEq L^−1^). (i and j) Water transparency to UV radiation (320 nm) (**i**) and PAR (400–700 nm) (**j**) expressed as the depth (m) to which 1% of subsurface irradiance penetrates. Lines are 20 year LOESS smoothed trends; bold indicates significant trends (P < 0.05, Mann-Kendall nonparametric trend test). Mann-Kendall Tau (τ), significance level (*P*), and number of years of data (n): annual air temperature τ = 0.05, *P* = 0.71, *n* = 27; spring air temperature τ = −0.02, *P* = 0.90, *n* = 27; spring wind speed τ = 0.42, *P* = 0.01, *n* = 22; precipitation τ = 0.31, *P* = 0.026, *n* = 27; NO_3_^−^ τ = −0.48, *P* = 0.003, *n* = 20; SO_4_^2−^ τ = −0.47, *P* = 0.004, *n* = 20; Giles [DOC] τ = 0.74, *P* < 0.001, *n* = 20; Lacawac [DOC] τ = 0.25, *P* = 0.13, *n* = 20; Giles a*320 nm τ = 0.42, *P* = 0.011, *n* = 20; Lacawac a*320 nm τ = 0.23, *P* = 0.17, *n* = 20; Giles pH τ = 0.92, *P* < 0.001, *n* = 21; Lacawac pH τ = 0.34, *P* = 0.025, *n* = 23; Giles ANC τ = 0.69, *P* < 0.001, *n* = 20; Lacawac ANC τ = 0.46, *P* = 0.006, *n* = 19; Giles 1%UV320 τ = −0.73, *P* < 0.001, *n* = 22; Lacawac 1%UV320 τ = −0.33, *P* = 0.034, *n* = 22; Giles 1%PAR τ = −0.59, *P* < 0.001, *n* = 27; Lacawac 1%PAR τ = −0.41, *P* = 0.003, *n* = 27.

There was no significant trend in either mean annual air temperature or spring (March – mid-July date of sampling) air temperature in the region during the 1988–2014 study period (Fig. 1a). There was a significant increase in spring (March – April) wind speed (Fig. 1b). These two different “spring” periods were chosen as those most likely to influence water temperatures in the epilimnion (air temperature in the whole ice-free period before the sampling), and hypolimnion (wind speed in March-April following ice-out, but before thermal stratification is established). Annual precipitation also increased significantly during this period (Fig. 1c), and atmospheric deposition of both SO_4_^2−^ and NO_3_^−^ decreased (Fig. 1d).

In-lake changes in Giles were greater than, but not opposite those in Lacawac. Average DOC concentrations and color (a*_320nm_) increased in both lakes but increases were statistically significant only in Giles where DOC concentration doubled (Fig. 1e,f). The increase in pH showed over a ten-fold decrease in hydrogen ion concentration in Giles and a smaller but significant trend in Lacawac (Fig. 1g). Both lakes experienced significant increases in acid neutralizing capacity (ANC), with Giles recovering from a negative alkalinity in the 1990s (Fig. 1h). Browning in both lakes was evident from significant declines in water transparency to both UV and PAR penetration during the study period (Fig. 1i,j). In Giles the 1% UV attenuation depth declined from over 10 m to about 2 m (Fig. 1i), and the 1% PAR attenuation depth declined from 23 m to less than 15 m (Fig. 1j). Declines in water transparency were more modest but also statistically significant in Lacawac (Fig. 1i,j).

**Figure 2:**
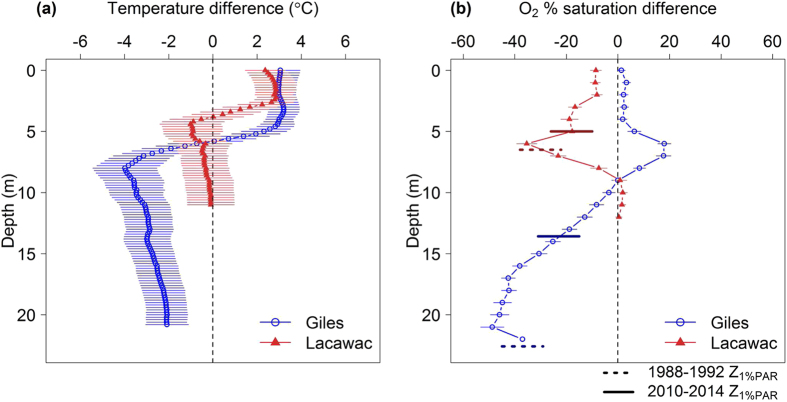
Differences between the averages (±S.E.M.) in O_2_ and temperature profiles from the first (1988–1992) to the last (2009–2014) five years of the study period. (**a**) Temperature difference (^o^C) versus depth (m). (**b**) O_2_ (percent saturation) difference versus depth (m). Thick horizontal lines are the compensation depths, estimated by 1% subsurface PAR. Note that the depth of peak loss in O_2_ is bracketed above and below by the historic (dashed line) and more recent (solid line) 1% PAR depths in both lakes.

**Figure 3:**
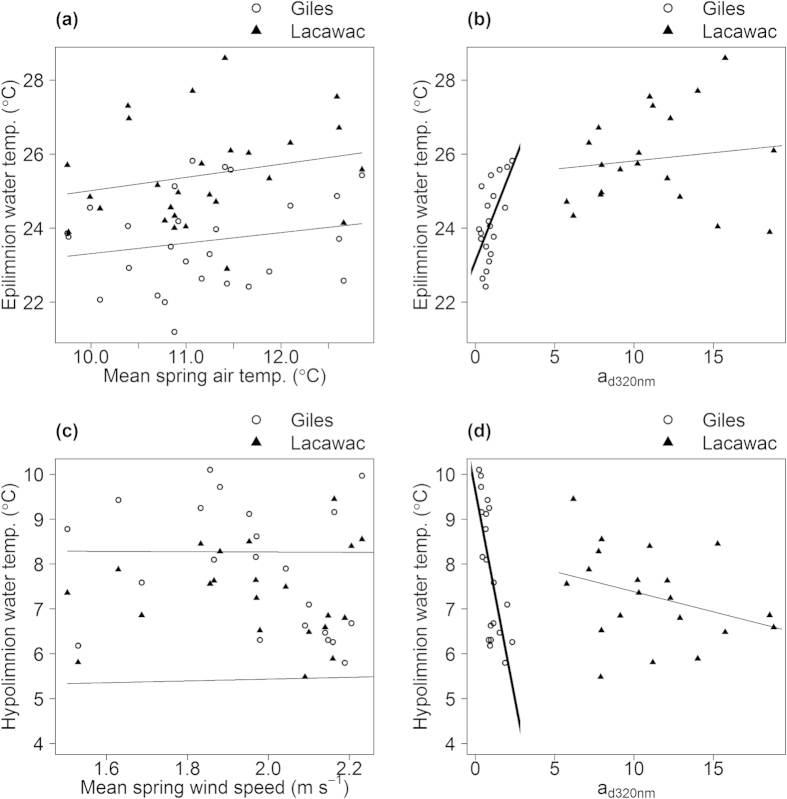
Relationships between water temperatures and air temperature, wind speed, and water transparency. The relationships between (**a**) July mean epilimnion water temperature and average spring (March – mid-July date of sampling) air temperature (Lacawac τ = 0.174, *P* = 0.21; Giles τ = 0.117, *P* = 0.40), and (**b**) July mean epilimnion water temperature and water transparency measured as a_d320nm_ and (Lacawac τ = 0.18, *P* = 0.28; Giles τ = 0.40, *P* = 0.01), and between (**c**) July mean hypolimnion water temperature and average spring (March-April) wind speed (Lacawac τ = −0.004, *P* = 1; Giles τ = −0.27, *P* = 0.09). Relationships between (**d**) July mean hypolimnion water temperature and water transparency measured as a_d320nm_ (Lacawac τ = −0.242, *P* = 0.14; Giles τ = −0.596, *P* < 0.001). Lines represent linear regression models with bold indicating significant trends (*P* < 0.05), Kendall non-parametric tests.

Water temperature and O_2_ showed complex, depth-specific changes during browning. Comparison of the average responses in the first versus last five years of the study period reveal that surface water temperatures have warmed by 2–3 °C in both lakes, while deeper waters cooled or showed no significant change depending on the lake and depth (Fig. 2a). In Lacawac the waters at intermediate depths trended toward cooling while the deeper waters in Giles cooled by as much as 4 °C (Fig. 2a). These depth specific changes in water temperature led to stronger thermal stratification in both lakes. Surface water temperatures were unrelated to mean air temperature during the months preceding sampling in both lakes (Fig. 3a), but were strongly related to water transparency in Lake Giles (Fig. 3b). Hypolimnetic temperatures were not related to spring wind speed in either lake, despite its increasing trend (Fig. 3c), but did show a strong decrease with decreasing water transparency in Lake Giles (Fig. 3d). Increases in wind speed would suggest the potential for greater wind-driven mixing and warmer hypolimnion temperatures; however, this relationship was not observed. Percent saturation of O_2_ declined at mid-water depths in Lacawac with the greatest decline at a depth of 6 m, while in Giles the decline was in deeper depths with the greatest decline at 21 m (Fig. 2b). The average (±S.E.M.) depth of O_2_ supersaturation (defined here by >110% saturation) in Lake Giles shallowed from 12.0 ± 0.61 m during 1988–1992 to 8.0 ± 0.42 m during 2010–2014. This led to opposite trends in O_2_ at intermediate depths between about 5–8 m in the two lakes wherein O_2_ declined in Lacawac and increased in Giles (Fig. 2b). These changes are likely related to the observed reductions in water transparency and a consequently shallower chlorophyll maximum.

**Figure 4:**
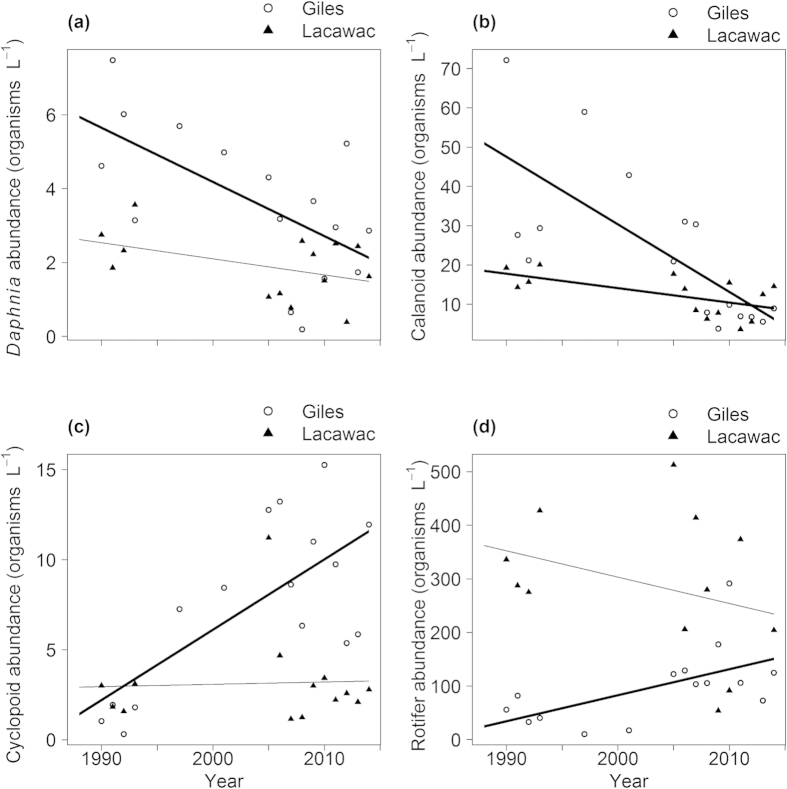
Interannual changes in the zooplankton. (**a**) The cladoceran *Daphnia*, a strong grazer on phytoplankton. (**b**) Calanoid copepods, omnivorous grazers on phytoplankton. (**c**) Predatory cyclopoid copepods. (**d**) Rotifers, a preferred prey of cyclopoid copepods. Lines are linear regressions; bold indicates significant trends (*P* < 0.05): Giles *Daphnia* τ = −0.43, *P* = 0.022, *n* = 16; Lacawac *Daphnia* τ = −0.19, *P* = 0.381, *n* = 14; Giles calanoids τ = −0.57, *P* = 0.003, *n* = 16; Lacawac calanoids τ = −0.43, *P* = 0.038, *n* = 14; Giles cyclopoids τ = 0.38, *P* = 0.043, *n* = 16; Lacawac cyclopoids τ = −0.03, *P* = 0.913, *n* = 14; Giles rotifers τ = 0.41, *P* = 0.038, *n* = 15; Lacawac rotifers τ = −0.30, *P* = 0.193, *n* = 12.

These altered vertical habitat gradients were accompanied by pronounced changes in the pelagic food web, particularly in Giles. Though data are limited to only a few years, extensive efforts to trap planktivorous juvenile (young-of-year, YOY) fish in Lake Giles in 1991 led to no captures, while catch per unit effort (CPUE) in the epilimnion of Lake Giles in the past 3 years averaged 5.5 individuals per 24 h trap period with a CPUE of up to 26 fish in a single trap. A similar increase was observed in YOY fish in Lacawac with an average CPUE of 1.6 and 5.0 in 1991 and 2014 respectively. The two dominant zooplankton grazers in Giles both showed substantial declines. The average May-August abundance of the cladoceran *Daphnia* in Giles declined to only half of its original numbers, and the calanoid copepods declined by five-fold (Fig. 4a,b). In contrast, the abundance of cyclopoid copepods and their small rotifer prey increased in Giles with the cyclopoids increasing by five-fold or more (Fig. 4c,d). In Lacawac only the calanoid copepods declined significantly and their decline was much less pronounced than in Giles (Fig. 4b). No significant trends in chlorophyll *a* concentration were observed in either Giles (τ = 0.28, *p* = 0.11, *n* = 18) or Lacawac (τ = −0.22, *p* = 0.26, *n* = 16) during the study period.

**Figure 5:**
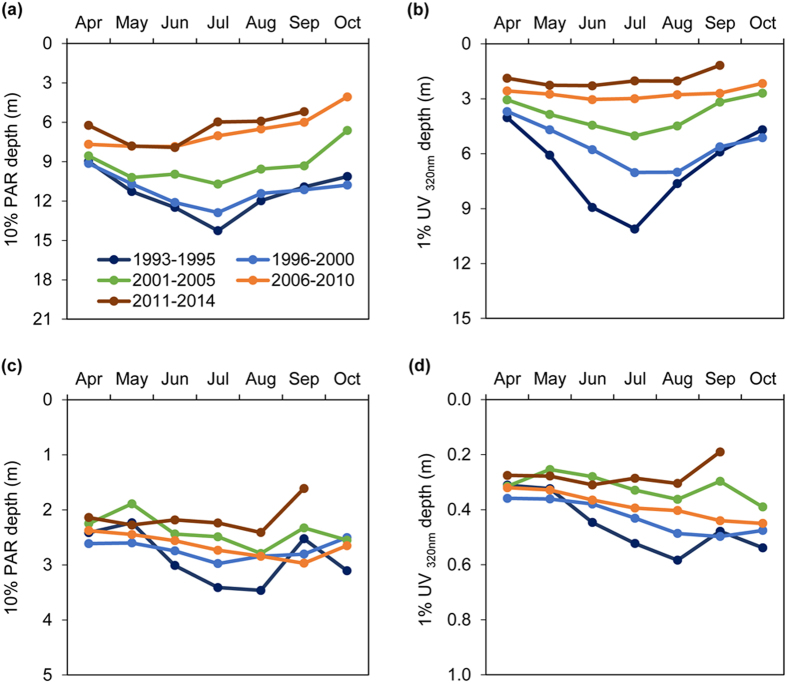
Seasonal changes in water transparency to UV and PAR. (**a**,**c**) Average depth to which 10% PAR (400–700 nm) penetrated in Giles (**a**) and Lacawac (**c**) during the designated time periods. (**b** and **d**) Average depth to which 1% 320 nm UV radiation penetrated in Giles (**b**) and Lacawac (**d**) during the designated time periods. Note the strong spring to mid-summer increases in water transparency in the 1990s that comprise the clear-water phases that are absent in more recent years. Note differences in the scales on the Y axes.

Monthly data on water transparency indicate strong changes in the phenology of both UV and PAR transparency in the two study lakes. Pronounced UV and PAR clear-water phases that were observed early in the study period in both lakes have disappeared in more recent years (Fig. 5). These clear-water phases were historically strongest in Giles where the measured 1% attenuation depth for potentially damaging 320 nm UV increased from 4 m in the spring to over 10 m by mid-summer, and the measured 10% PAR attenuation depth increased from 9 m to 14 m during this same period (Fig. 5a,b). Similar but less pronounced changes in these UV and PAR clear-water phases were observed in Lacawac (Fig. 5c,d).

## Discussion

Our long-term observations are generally consistent with short-term experimental, comparative, and modeling studies, and with emerging evidence for the central importance of shading effects during browning^9^. These shading effects are particularly important in small lakes that are sheltered by the surrounding forest and thus minimally influenced by wind^18^,^19^, as are Lacawac and Giles. Evidence includes decreases in UV and PAR transparency, changes in thermal structure with no increase in regional air temperature, increases in O_2_ depletion, a shift in the O_2_ maximum to a shallower depth in Giles, and declines in the two primary zooplankton grazers. In contrast, increases in YOY fish and the addition of a trophic level in the zooplankton community are more consistent with either a resource subsidy hypothesis or the release of YOY fish from low pH or high UV exposure^12^. Regardless of the underlying mechanisms, these ecological consequences of browning have important implications for critical ecosystem services. The changes in food web structure suggest an increase in the trophic transfer to higher trophic levels such as fish and are relevant to fishery productivity^14^. The decreases in water transparency to UV and PAR decrease the potential for solar disinfection of parasites and pathogens^20^. The increase in DOM itself can increase the production of carcinogenic disinfection byproducts in drinking water as well as the cost of water purification for municipal use^21^. Browning may also influence the temperature and O_2_ dependent production of CO_2_ and CH_4_ from lake sediments and the role of inland waters as a globally important source of greenhouse gases^22^. Browning may either increase^23^ or decrease^24^ the sequestration of greenhouse gases depending on the balance between mineralization of DOC to carbon dioxide and burial of organic carbon in the sediments. Colder temperatures and anoxia in the hypolimnion may increase deposition of fixed carbon in the sediments by reducing decomposition rates, but also increase the production of methane, which is a much more powerful greenhouse gas than is the carbon dioxide produced under well oxygenated conditions. Even short-term extreme precipitation events can induce browning that reduces water clarity, shifts lake metabolism from autotrophy to heterotrophy^25^, alters vertical distribution of important zooplankton grazers^26^, and depletes O_2_ leading to hypoxia or anoxia^2^,^25^.

The stronger long-term trends in Giles versus Lacawac are consistent with the concept that less transparent, higher DOM lakes are more resistant to browning while clear-water lakes are more sensitive sentinels of ecosystem level responses to browning^7^,^13^. The stronger thermal stratification in both lakes is likely due to an increased absorption of sunlight in the browner surface waters, and a consequent reduction in heat energy transferred to deeper waters^13^. Mechanistic models have clearly demonstrated the potential for DOM-induced changes in transparency to alter thermal structure in lakes to the extent observed in our two study lakes including warmer surface water temperatures and cooler deeper waters, as well as stronger effects in more transparent lakes^13^. In very clear lakes like Giles, these effects are particularly pronounced due to the strong changes in water transparency in response to increases in DOM. PAR penetrated to the bottom of Giles (1% of surface irradiance was 23 m, Fig. 1j) early in the study period, but it often penetrated less than 15 m later in the study period, thus severely reducing the potential for solar heating of deeper waters in more recent years.

The changes in the phenology of water transparency were not predicted by shorter-term studies of browning and signal important changes in lake ecosystem structure and function. Zooplankton grazers such as *Daphnia* play an important role in grazing down spring phytoplankton blooms, leading to a seasonal increase in water transparency. Thus the disappearance of the PAR clear-water phase during browning is consistent with the observed decline in zooplankton grazers. The disappearance of the UV clear-water phase likely signals a change in DOM processing by photodegradation^15^. Breakdown of DOM by sunlight has recently been demonstrated to be more important in high latitude lakes than previously appreciated^24^, and is known to be responsible for a majority of the seasonal UV clear-water phases observed in the past in Lake Giles^15^.

It is critical to note that we have examined only two lakes, and the responses of other lakes to browning are likely to vary as a function of not only transparency, but lake and catchment geomorphology, geology, local hydrology, atmospheric deposition, and other environmental conditions. However, small lakes like these study lakes dominate numerically on a global scale and are critical to the processing and fate of organic matter^27^. Browning may also have consequences for larger lakes and coastal ocean waters. In the Arctic thawing permafrost is releasing large amounts of biolabile and photolabile DOM to inland and coastal waters and altering underwater UV environments as well as greenhouse gas production^24^. Mesocosm experiments in coastal ocean waters have demonstrated that O_2_ depletion can cause P regeneration from the sediments as well as generate increases in DOM, leading to positive feedbacks that are similar to those described in lakes^2^,^28^. Novel UV-based optical approaches coupled with remote sensing can identify terrestrially derived lignin components of DOM and enable visualization of the influence of browning on regions as expansive as the Arctic Ocean^29^. These kinds of advanced sensor approaches will be critical to resolving the extent to which the ecological consequences and threat to ecosystem services observed in browning lakes are also a threat to coastal ocean ecosystems and global greenhouse gas production. While the greater importance of wind-driven mixing and a greater buffering capacity will likely mask the effects of browning in many larger lakes and oceans, recovery from acidification as well as interactions with iron may enhance the browning and hence increase thermal stratification of smaller inland lakes.

Our data indicate that the widespread increases in surface water temperatures and thermal stratification that have been associated with climate warming in lakes and oceans^30^,^31^,^32^,^33^ may be augmented by increases in precipitation-induced transport of terrestrially-derived DOM. Thus in regions where lakes and coastal oceans are browning, both the wetter as well as the warmer conditions associated with climate change may combine to provide a double-edged sword that more strongly increases the strength of thermal stratification and potential for associated threats to ecosystem services across these inland and coastal ecosystems.

## Methods

Water samples were collected from a single depth in the epilimnion using a Van Dorn bottle. All variables except zooplankton were measured from a single sample collected between mid-July and mid-August, during the period of maximum thermal stratification. Strength of stratification was calculated by subtracting the average hypolimnion temperature from the average epilimnion temperature. In both lakes, a period of peak stratification (±10%) typically extends over a period of 7–11 weeks from early to mid-July into late August or early September. Zooplankton densities are annual averages from May to August. For measurements of DOC and absorbance, samples were filtered through pre-combusted 0.7 μm glass-fiber filters shortly (generally within 48 h) after collection. For DOC, samples were analyzed through high temperature oxidation in a TOC analyzer. Decadal dissolved absorbance at 320 nm was measured using a spectrophotometer and converted to Naperian dissolved absorption coefficients (a_d_) using the following relationship^34^:


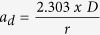


Where D is the raw absorbance value at 320 nm and r is the path length (m) of the quartz cuvette. The a*320 nm was calculated as the absorbance at 320 nm per unit DOC (mg L^−1^). Raw water samples were analyzed for pH using an Orion pH meter equipped with a Ross epoxy-body combination electrode, calibrated at pH 7.00 and 4.00 using commercial pH buffers. Following pH measurements, ANC was determined through potentiometric titration with dilute HCl (1988–1993) or H_2_SO_4_ (0.01 N for Giles and 0.1 N for Lacawac). Titration points between pH 4.4 and 3.7 were plotted after Gran transformation, and ANC was calculated as μEq L^−1^. For Chl *a* analysis, water samples were filtered through a glass fiber filter and immediately frozen. Filters were extracted for 24–48 h in 90% alkaline acetone:methanol (1990–1993, 2010-present, Lacawac 1995) and analyzed using a fluorometer. Several minor variations on this general method were used over the course of the study. Samples from 1990–1993 were extracted in the refrigerator. In Giles 1995 and 1996 and Lacawac 1994, samples were placed in 90% ethanol solution, heated to 100 °C and extracted in the freezer for 24 h prior to analysis in a spectrophotometer. A heating step (65 °C for two minutes) was also employed at the midpoint of a 48 h freezer extraction from 2010–2014^35^. Following fluorometric analysis, samples were acidified with 0.03N HCl to differentiate Chl-*a* from phaeophytin. Direct intercomparisons of the acetone and acetone/methanol solvents for Chl extraction and the opposite (non-significant) Chl *a* trends in the two lakes indicate no methodological artifacts in the Chl *a* data.

Temperature, O_2_ and light were taken *in situ* at a 1 meter depth or higher resolution. Temperature (1988–1992) and O_2_ (all years) were measured at 1-meter intervals using a YSI oxygen meter. Temperature from 1993–2014 was measured using a submersible radiometer. The difference in both temperature and O_2_ (in percent saturation) at each meter depth was calculated using the five most recent years (2010–2014) and the five earliest years (1988–1992). UV and PAR light profiles and the measured depth to which 1% of surface irradiance penetrated were collected using one of three methods, including a Li-Cor sensor (PAR; 1988–1993), PUV radiometer (PAR and UV; 1993–2003) or a BIC radiometer with a deck cell (PAR and UV; 2003–2014).

Zooplankton densities are from integrated net tows taken through the whole water column. In Giles, the zooplankton community consists of *Daphnia catawba*, *Cyclops scutifer*, calanoid copepods (87% *Leptodiaptomus minutus*, 12% *Aglaodiaptomus spatulocrenatus*), and rotifers (29% *Keratella spp*., 27% *Kellicottia spp.,* 26% *Polyarthra spp., and 11% Gastropus spp.*). In Lacawac, there are two species of *Daphnia* (72% *D. ambigua* and 27% *D. catawba*), a single species of calanoid copepod (*L. minutus*), two species of cyclopoid copepods (64% *C. scutifer* and 36% *Mesocyclops edax*), and rotifers (35% *Keratella spp.,* 30% *Polyarthra spp.,* and *18% Kellicottia spp.*). No significant trends in the relative abundance of the two cyclopoid copepod species in Lacawac were observed during the study period. While some calanoid copepods feed on rotifers^36^,^37^, the dominant rotifer genera in these lakes (*Keratella*, *Kellicottia*, and *Polyarthra*) have effective defense or avoidance responses to Diaptomid predators and are not the preferred prey of the very small body sized dominant calanoid, *L. minutus*, in our two study lakes. From 1990–1993 and 2005–2008 *Daphnia* were collected with a 202 μm bongo net and counted in a Bogorov chamber, except in Lacawac in 2008 where a Wisconsin-style 48 μm net and Sedgewick Rafter chamber were employed. In 1997, 2001, 2009–2011, and 2014 *Daphnia* were collected in a 48 μm Wisconsin-style bongo net and counted in a Bogorov chamber. Intercomparison of the nets demonstrated similar high collection efficiencies for the two mesh sizes. For most years, both calanoid and cyclopoid copepods, as well as rotifers were collected with a 48 μm net and counted in a Sedgewick Rafter chamber. Exceptions include 1990–1993 when female cyclopoid copepods were collected with a 202 μm Wisconsin-style bongo net and counted in a Bogorov chamber; and 2012 and 2013, when all copepods and *Daphnia* were collected using a 153 μm Wisconsin-type bongo net and counted in a Bogorov chamber. Copepod analyses include males, females and copepodids. *Daphnia* counts include juveniles and adults (not neonates). Total rotifer abundance includes all individual rotifers with the exception of the genus *Conochilus*, which can be found both as a colony and individually, and was therefore excluded from analysis.

Data on YOY larval fish represent 24 h trapping efforts using clear plastic replicate (2 separate sides) minnow traps suspended at a depth of 1.5 m, deployed between 15:00–17:00 h, and expressed as CPUE. The traps used in the 1990’s and the 2010’s were the same identical traps. YOY trapping in Lacawac was conducted on 31 May – 1 June, 13–14 July, and 25–26 August, 1991 for a total of 12 traps deployed over each 2 day period (6 traps per 24 hour period that were emptied and immediately redeployed, with a similar effort in Lake Giles). In 2012 trapping occurred in Lake Giles on three different dates (17 June, 3 and 20 July) with six traps used per date. In 2013 only two traps were used per date, but the trapping was done on 12 different dates from early July through mid-August (2, 3, 9, 12, 16, 19, 23, 25, 30 July and 2, 6 and 8 August). In 2014 trapping in both Giles and Lacawac occurred on a single date (3, 13 July, respectively) with two traps per lake. The species captured in Lake Giles from 2012–2014 were approximately 90% *Micropterus salmoides* on all trap dates with up to 10% of the individuals being *Lepomis spp.* Fish species in Lacawac during the 1991 trapping included *Perca flavenscens* as the only species captured 31 May – 1 June, 50% *Lepomis spp.* and 50% *P. flavenscens* during the 13–14 July trapping, and only *Lepomis spp.* on 25–26 August. In 2014, the species present in Lacawac were largely *M. salmoides* and *M. dolomieui* with approximately 15% of the individuals captured being *Lepomis spp*.

Meteorological data on minimum and maximum daily air temperature and precipitation were based on daily data obtained from the National Oceanic and Atmospheric Administration National Climate Data Center using the nearest station (<15 km to the two lakes) located in Hawley, Pennsylvania , USA (GHCND:USC00363758). Mean daily air temperature was calculated from averaging daily minimum and daily maximum air temperature. Annual mean air temperature was the previous 365-day average of the temperature profile, inclusive of the sampling day (roughly 16 July – 15 July of the following year). Spring mean air temperature was calculated from the average 01 March to day of sampling for each year. Data for annual wet deposition of SO_4_^2−^ and NO_3_^−^ were downloaded from the National Atmospheric Deposition Program monitoring site at the nearest station (40 km to Lacawac, 23 km to Giles) located in Milford, Pennsylvania, USA (PA72^38^). Wet deposition data prior to 1994 were excluded from our analysis due to contamination in the samples leading to overestimation, and are not comparable to deposition data after 1994. Wind speed data (1993–2014) were collected from an on-lake buoy at Lacawac using an anemometer at 2 m height (maintained by B.R. Hargreaves, Lehigh University). Spring wind speed was calculated from the average 01 March – 30 April daily wind speed for each year.

## Statistical Analysis

Mann-Kendall non-parametric trend tests were used for temporal trends of all variables^39^,^40^. Significance of trends were considered at the *P* < 0.05 level. For temporal trend models, LOESS 20-moving averages were used for most variables. For zooplankton trends, linear models were used instead due to many years of missing data and the high variability (Mann-Kendall trend tests were still used to assess significance). Kendall univariate tests were run to assess relationships between spring air temperature and epilimnion temperature, spring wind speed and hypolimnion temperature, and a_d320nm_ and both epilimnion and hypolimnion temperature (independently). All statistical analyses and models were run under the “Kendall”^41^ and “wq”^42^ packages and were completed in R^43^ (version 3.1.1).

## Additional Information

**How to cite this article**: Williamson, C. E. *et al.* Ecological consequences of long-term browning in lakes. *Sci. Rep.* **5**, 18666; doi: 10.1038/srep18666 (2015).
